# Is working in a cold environment associated with musculoskeletal complaints 7–8 years later? A longitudinal analysis from the Tromsø Study

**DOI:** 10.1007/s00420-020-01606-6

**Published:** 2020-11-23

**Authors:** Erlend Hoftun Farbu, Anje Christina Höper, Tormod Brenn, Morten Skandfer

**Affiliations:** 1 Department of Community Medicine, UiT-The Arctic University of Norway, Tromsø, Norway; 2 Department of Occupational and Environmental Medicine, University Hospital of North Norway, Tromsø, Norway

**Keywords:** Musculoskeletal pain, Musculoskeletal complaints, Cold environment, Epidemiology, Cold temperature

## Abstract

**Objective:**

Exposure to a cold environment at work is associated with a higher prevalence of musculoskeletal pain and chronic pain in cross-sectional studies. This study aims to determine the association between working in a cold environment ≥ 25% of the time and musculoskeletal complaints (MSC) 7–8 years later.

**Methods:**

We followed participants from the sixth survey (Tromsø 6, 2007–2008) to the seventh survey (Tromsø 7, 2015–2016) of the Tromsø Study. Analyses included 2347 men and women aged 32–60 years who were not retired and not receiving full-time disability benefits in Tromsø 6. Three different binary outcomes were investigated in Tromsø 7: any MSC, severe MSC, and MSC in ≥ 3 anatomical regions. We excluded participants with severe MSC, MSC in ≥ 3 regions, or missing values in Tromsø 6. The association between working in a cold environment and future MSC were examined using Poisson regression and adjusted for age, sex, number of moderate MSC, education, physical activity at work, smoking status, body mass index, and self-reported health in Tromsø 6.

**Results:**

258 participants reported to work in a cold environment ≥ 25% of the time in Tromsø 6. They had an increased risk of having any MSC in Tromsø 7 (incidence rate ratio 1.15; 95% confidence interval 1.03–1.29). There was no significantly increased risk of severe MSC or MSC in ≥ 3 regions.

**Conclusion:**

Working in a cold environment was associated with future MSC, but not with future severe MSC or future MSC in ≥ 3 regions.

**Electronic supplementary material:**

The online version of this article (10.1007/s00420-020-01606-6) contains supplementary material, which is available to authorized users.

## Introduction

Even moderately cold temperatures can cause stress to the human body and increase mortality (Gasparrini et al. 2015). Exposure to a cold environment at work has been suggested as a risk factor or aggravator of different health complaints, such as musculoskeletal pain and symptoms from skin, the respiratory system, and the cardiovascular system (Makinen and Hassi 2009).

A cold working environment is defined as a temperature below 10 °C (ISO 15743:2008 Ergonomics of the thermal environment—cold workplaces—risk assessment and managment 2008), but cold stress might be present even at higher temperatures in the workplace (Bang et al. 2005). In addition, ambient temperature is only one of many factors that determine a worker’s heat loss. Clothing, air movement, contact with cold surfaces and liquids, and the amount of heat produced by the work can also have an impact on a worker’s thermal balance and thereby possibly lead to health complaints.

Low back and neck pain are a major cause of disability-adjusted life years (Murray et al. 2012). Furthermore, chronic pain is strongly associated with future disability pension, due to both musculoskeletal and other disorders (Haukka et al. 2015; Saastamoinen et al. 2012). Several cross-sectional studies have found that working in a cold environment or feeling cold is associated with a higher prevalence of pain among slaughterhouse, construction, seafood industry, and storehouse workers, as well as in the general population. The association has also been found for musculoskeletal locations such as the wrist, back, neck, and shoulder (Aasmoe et al. 2008; Dovrat and Katz-Leurer 2007; Farbu et al. 2019; Pienimaki 2002; Skandfer et al. 2014). In a cohort of 134,754 male Swedish construction workers, there was a higher prevalence of musculoskeletal pain in geographical regions with lower mean temperatures (Burstrom et al. 2013). Some studies have reported a higher incidence of tendinopathies and associated disorders in colder environments (Kurppa et al. 1991; Milgrom et al. 2003). However, there is a need for more prospective studies investigating exposure to a cold environment at work as a risk factor for musculoskeletal pain.

Our previous cross-sectional study from the sixth survey of the Tromsø Study (Tromsø 6) found that working in a cold environment ≥ 25% of the time was associated with chronic pain lasting 3 months or longer (Farbu et al. 2019). In the consecutive, seventh survey of the Tromsø Study (Tromsø 7) the questions concerning chronic pain and anatomical sites were replaced with a computerised system, in which participants pointed and clicked on a digital mannequin to show affected sites, combined with questions. Thus, answers in Tromsø 6 and Tromsø 7 are not directly comparable. Therefore, the outcomes in this prospective analysis are based on another set of questions that were phrased identically in both surveys. However, as these questions assessed both pain and/or stiffness in the same question, we termed it “musculoskeletal complaints” (MSC).

This study aims to determine the association between working in a cold environment ≥ 25% of the time and MSC 7–8 years later.

## Methods

### Population: the Tromsø Study

**Fig. 1:**
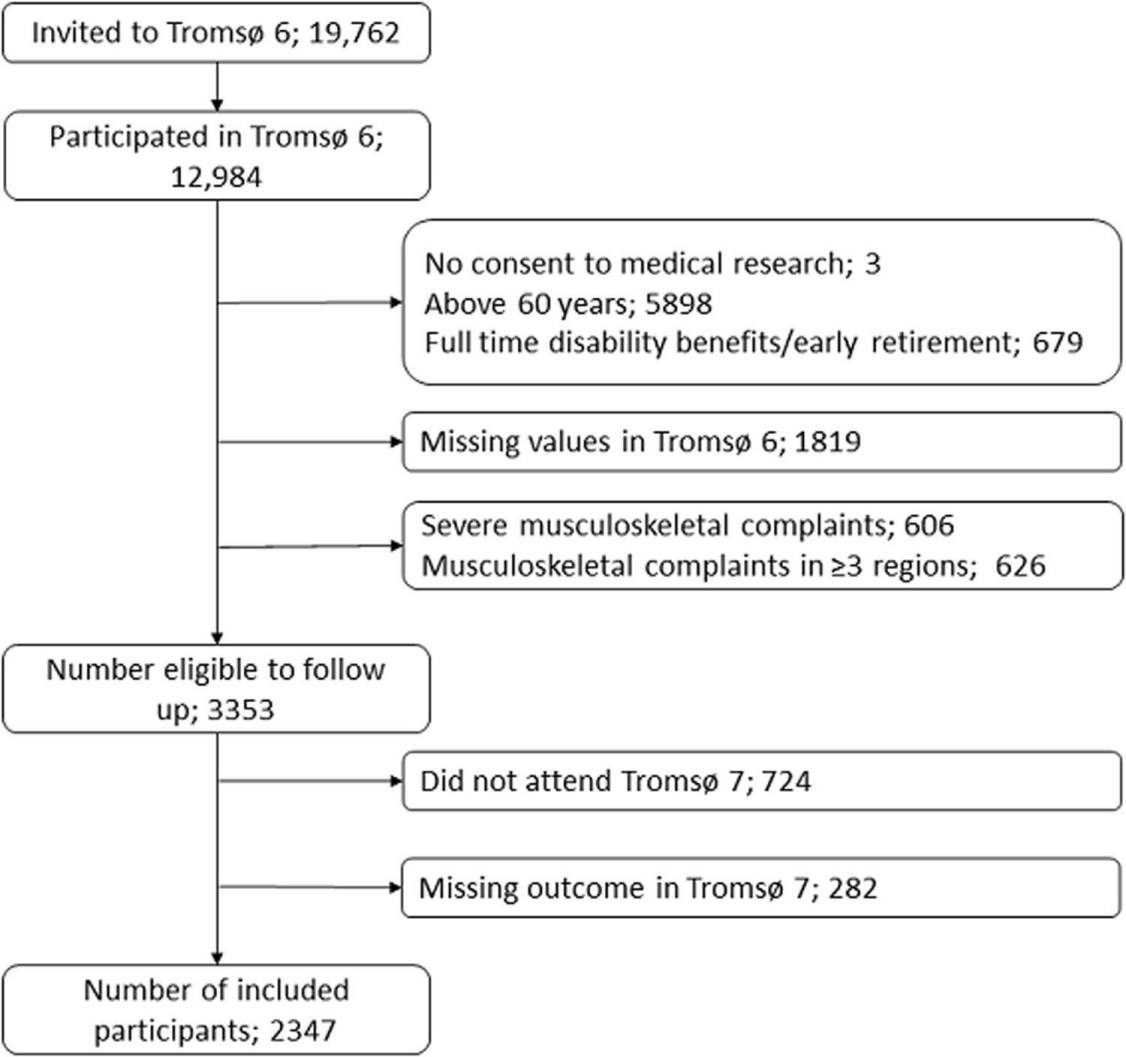
Flow chart presenting number of subjects invited to Tromsø 6, those who participated in Tromsø 6 and in Tromsø 7, and those excluded and included in the present analysis

The Tromsø Study is a prospective cohort study consisting of seven surveys carried out from 1974 to 2016. We used the data from Tromsø 6 (2007–2008) as the baseline and that from Tromsø 7 (2015–2016) as follow-up (Jacobsen et al. 2012). The surveys consist of a physical examination and questionnaires. As the risk of MSC is likely to decrease after retirement, we excluded all participants who were retired, older than 60, or receiving a fulltime disability pension at the time of Tromsø 6 (Neupane et al. 2018). Finally, we excluded all participants with missing values in Tromsø 6 (Fig. 1). The Regional Committee of Research Ethics approved Tromsø 6 and 7 and this particular analysis.

### Exposure and confounders

The question “Do you work outdoors or in cold buildings (e.g. storage/industry buildings) at least 25% of the time?” (Yes/No) from Tromsø 6 was the exposure of interest. Tromsø has a coastal climate; the outdoor temperature is below 10 °C for most of the year and seldom falls below − 10 °C [Weather statistics for Tromsø observation site, Tromsø (Troms) 2018]. In Tromsø 7 there was no measure for cold exposure.

Information on age and education was taken from Tromsø 6. The degree of physical activity at work was assessed with the question “If you have paid or unpaid work, which statement describes your work best?”, with four response alternatives: mostly sedentary, requires a lot of walking, requires a lot of walking and lifting, and heavy manual labour. Smoking status was categorised as current, former, and never smoker. Body mass index (BMI) was calculated from height and weight measures at the Tromsø 6 physical examination. Self-reported health was assessed with the question “How do you in general consider your own health to be?”, with five response alternatives: excellent, good, neither good nor bad, bad, and very bad. Due to few respondents reporting bad and very bad, the categories were merged with “neither good nor bad”.

### Outcomes

In both Tromsø 6 and Tromsø 7, information on MSC was collected with the question “During the last year have you been affected by pain and/or stiffness in muscles or joints lasting at least 3 months?” with a list of six different anatomical regions: neck or shoulder, upper back, lumbar back, hip or leg, arm or hand, and other. For each site there were three alternatives: no, moderately, or severely. We investigated three different binary outcomes: any MSC, severe MSC, and MSC in ≥ 3 regions. Those reporting moderate or severe MSC at one or more regions were categorised as having “any MSC”. Participants who reported severe MSC at any of the six regions were categorised as “severe MSC”. For the third binary outcome, we counted the number of regions with MSC, regardless of severity, and categorised them into 0–2 regions and ≥ 3 regions. We excluded all those who reported severe MSC or MSC in ≥ 3 regions in Tromsø 6.

### Statistical analyses

Pearson chi-square was used to test differences in prevalence, and *t *test was used for age. We used Poisson regression with robust variance to perform three different analyses for the three binary outcomes; any MSC, severe MSC, and MSC in ≥ 3 regions. Poisson regression is recommended when analysing binary outcomes with high prevalence. The effect estimate is incidence rate ratio (IRR) and can be interpreted as relative risk (Zou 2004). All statistical analyses were performed in Stata MP 15.

### Sensitivity analysis

A large proportion of participants were excluded due to missing values in Tromsø 6 (Fig. 1). As this could introduce bias, we performed multiple imputation with chained equations. We included the original questions about MSC and all the variables included in the main analysis. To increase the predictive power, we included dichotomous questions about chronic pain from Tromsø 6 and Tromsø 7, as well as pain sites from Tromsø 7. Due to perfect prediction, we used the augment option and imputed 100 datasets. To make the IRRs from the imputed regression models comparable to the IRRs from the main analysis, we included an interaction term between having severe MSC or MSC in ≥ 3 regions in Tromsø 6 and working in a cold environment ≥ 25% of the time in the regression analysis on the imputed datasets.

To investigate possible confounding by occupational factors, we conducted sensitivity analyses using occupational codes obtained from the NAV State Register of Employers and Employees (NREE), which is administrated by Statistics Norway. Employers are required to register all those employed for at least 7 days and who will likely have an average of more than 4 h’ work per week in the NREE. Each employee is registered with an industrial classification code using the Norwegian coding system, STYRK-98. The 4 first digits of this system are similar to those in the International Standard Classification of Occupations 88. We used the unique 11-digit identification number assigned to all individuals living in Norway to link the NREE with the data from the Tromsø Study. The NREE was not complete at the time of Tromsø 6; therefore, we restricted sensitivity analyses to the subsample of participants with an existing occupational code in the NREE in 2007. For those missing a code in 2007 but with an existing code in 2008, we used the one from 2008. To assess the possible confounding effect of occupation on the association between working in a cold environment and MSC, we ran three different logistic regression analyses: (1) a model identical to that in the main analysis; (2) a model adjusted for the 10 major occupational groups in the International Standard Classification of Occupations 88; (3) a mixed-effects logistic model with a random intercept for each 4-digit occupational code.

## Results

Of the 2347 participants, 258 reported working in a cold environment ≥ 25% of the time in Tromsø 6. The latter participants reported more moderate MSC in Tromsø 6, had less education, were more physically active at work, were more often smokers or former smokers, and a higher BMI than those working in a cold environment < 25% of the time. They also had poorer self-reported health. There were no significant differences in age at the time of Tromsø 6 between those working in a cold environment ≥ 25% of the time and those who did not (Table 1).

**Table 1:** Characteristics of the study population in Tromsø 6 (baseline)

	Working in a cold environment ≥ 25% of the time				
	No, *n* = 2089		Yes, *n* = 258		*t*/*χ*^2^
	*n*	%	*n*	%	*p*
Age^a^	47.1	6.9	46.7	6.9	0.396
Sex					
Female	1041	50	52	20	
Male	1048	50	206	80	< 0.001
Number of moderate musculoskeletal complaints					
0	1260	60.3	123	47.7	
1	483	23.1	66	25.6	
2	346	16.6	69	26.7	< 0.001
Education					
Primary/secondary	154	7.4	61	23.6	
Technical school	370	17.7	107	41.5	
High school	196	9.4	37	14.3	
College/university less than 4 years	559	26.7	41	15.9	
College/university 4 years or more	810	38.8	12	4.7	< 0.001
Physical activity at work					
Mostly sedentary work	1387	66.4	33	12.8	
Work that requires a lot of walking	474	22.7	71	27.5	
Work that requires a lot of walking and lifting	223	10.7	125	48.5	
Heavy manual labour	5	0.2	29	11.2	< 0.001
Smoking status					
Current	303	14.5	57	22.1	
Former	745	35.7	101	39.1	
Never	1041	49.8	100	38.8	0.001
Body mass index					
Under and normal weight (< 25 kg/m^2^)	892	42.7	85	32.9	
Overweight (≥ 25 and < 30 kg/m^2^)	886	42.4	131	50.8	
Obese (≥ 30 kg/m^2^)	311	17.9	42	16.3	0.01
Self-reported health					
Bad/very bad/neither good nor bad	231	11.1	40	15.5	
Good	1193	57.1	171	66.3	
Excellent	665	31.8	47	18.2	< 0.001

^a^Numbers are mean and standard deviation for age

### Musculoskeletal complaints

In Tromsø 7, those who reported working in a cold environment ≥ 25% of the time in Tromsø 6 had a higher prevalence of both moderate and severe MSC (Table 2). The prevalence of participants with MSC in 1–2 and ≥ 3 regions was higher in the exposed group. These differences were evident among those who had no MSC in Tromsø 6, while there were no significant differences among those reporting moderate MSC in Tromsø 6.

**Table 2:** Participants having no or moderate musculoskeletal complaints (MSC) in Tromsø 6, and severity of MSC and MSC in 0, 1–2, or ≥ 3 anatomical regions in Tromsø 7

Tromsø 7	No MSC in Tromsø 6					Moderate MSC in Tromsø 6				
	Working in a cold environment ≥ 25% of the time					Working in a cold environment ≥ 25% of the time				
	No *n* = 1260		Yes *n* = 123		*χ*^2^	No *n* = 829		Yes *n* = 135		*χ*^2^
	*n*	%	*N*	%	*p*	*n*	%	*n*	%	*p*
Severity of MSC										
No	697	55.3	53	43.1		251	30.3	39	28.9	
Moderate	500	39.7	62	50.4		471	56.8	78	57.8	
Severe	63	5.0	8	6.5		107	12.9	18	13.3	
					0.034					0.947
Number of MSC										
0	697	55.3	53	43.1		251	30.3	39	28.9	
1–2	420	33.3	48	39.0		364	43.9	61	45.2	
≥ 3	143	11.4	22	17.9		214	25.8	35	25.9	
					0.017					0.943

Those working in a cold environment ≥ 25% of the time had a significantly increased risk of any MSC in Tromsø 7, after adjustment for age, sex and number of moderate MSC in Tromsø 6 [IRR 1.13; 95% confidence interval (CI) 1.02–1.25] (Table 3). This association was slightly stronger after further adjustment for education, physical activity at work, smoking, BMI, and self-reported health in Tromsø 6 (IRR 1.15 95% CI 1.03–1.29).

**Table 3:** Incidence rate ratio’s (IRR) for any musculoskeletal complaints (MSC), severe MSC, and MSC in ≥ 3 anatomical regions in Tromsø 7

	Working in a cold environment < 25% of the time *n* = 2089		Working in a cold environment ≥ 25% of the time *n* = 258						
			Crude^a^				Fully adjusted model^b^		
	*n*	IRR	*n*	IRR	CI		IRR	CI	
Any MSC	1141	Ref	166	1.13	1.02	1.25	1.15	1.03	1.29
Severe MSC	170	–	26	1.14	0.76	1.70	0.95	0.60	1.48
MSC in ≥ 3 anatomical regions	357	–	57	1.27	0.98	1.64	1.11	0.83	1.49

^a^Adjusted for age, sex, and number of moderate MSC in Tromsø 6

^b^Adjusted for age, sex, number of moderate MSC, education, physical activity at work, smoking status, body mass index, and self-reported health in Tromsø 6

In the model using severe MSC as an outcome, those working in a cold environment ≥ 25% of the time had no significantly increased risk of MSC after adjustment for age, sex, and number of moderate MSC in Tromsø 6 (Table 3).

The risk of MSC in ≥ 3 regions was higher for those working in a cold environment ≥ 25% of the time in the model adjusted for age, sex, and number of moderate MSC in Tromsø 6 (IRR 1.27; 95% CI 0.98–1.64). However, the association was not significant, and further adjustment attenuated the association (IRR 1.11; 95% CI 0.83–1.49).

The results from the analysis using imputed data gave similar results. In the full model, those working in a cold environment ≥ 25% of the time had an increased risk of any MSC (IRR 1.12; 95% CI 1.03–1.22) and no increased risk of severe MSC (IRR 0.77; 95% CI 0.69–1.38) or MSC in ≥ 3 regions (IRR 1.08; 95% CI 0.88–1.32) (Supplementary Table 1).

### Sensitivity analyses with occupational codes

The logistic regression model that adjusted for the 10 major occupational groups did not substantially alter the strength of the association when all other covariates were included, nor did the mixed-effects model with a random intercept for each 4-digit occupational code (Supplementary Table 2). Supplementary Table 3 shows the different occupations in Tromsø 6 for those working in a cold environment ≥ 25% of the time.

## Discussion

### Key findings

This is the first prospective study of working in a cold environment as a risk factor for future MSC in the general working population. Those working in a cold environment ≥ 25% of the time had a significantly increased risk of experiencing any MSC with a duration of ≥ 3 months 7–8 years later. This association remained significant even after adjustment for baseline characteristics of age, sex, number of moderate MSC, education, physical activity at work, smoking, BMI, and self-reported health in Tromsø 6. The risk of severe MSC or MSC in ≥ 3 regions was not significantly higher for those working ≥ 25% of the time in a cold environment.

One previous study found an increased incidence of Achilles paratendinitis among recruits who completed their basic training in winter compared to summer (Milgrom et al. 2003). Another study of meat-house workers noted that the only noticeable difference between two groups with different incidences of tenosynovitis was a colder workplace environment (Kurppa et al. 1991). Other cross-sectional studies have found a higher prevalence of musculoskeletal pain among workers exposed to a cold environment. The studied populations were storehouse workers, construction workers, mine workers and seafood industry workers, and the general working population (Aasmoe et al. 2008; Burstrom et al. 2012; Dovrat and Katz-Leurer 2007; Farbu et al. 2019; Skandfer et al. 2014). In our previous study, we found an association between working in a cold environment and chronic pain at ≥ 3 anatomical sites (Farbu et al. 2019), but in the current study, we did not find any significant increased risk of MSC in ≥ 3 regions. The higher resolution of the outcome measure in our previous study could explain some of this difference, as the previous study investigated 14 different sites with chronic pain versus 6 regions with MSC in the current study, which means that a participant with pain in the neck, shoulder, and arm would have been classified differently in the two studies. However, since the outcomes in this study concern how much the participants are “affected by pain and/or stiffness”, and we do not know how many have stiffness without pain, direct comparison with our earlier research is precarious.

Among participants without MSC in Tromsø 6, those working ≥ 25% of the time in a cold environment had a significantly higher prevalence of MSC in Tromsø 7. However, there was no such difference among those with moderate MSC in Tromsø 6. This indicates that working in a cold environment could contribute to developing MSC, but not aggravate already existing MSC. This is consistent with the lack of significant associations for severe MSC and MSC in ≥ 3 regions. One explanation could be that those working in a cold environment are more prone to transient MSC, like tendinopathies, which often have quite a good prognosis even if left untreated (Smidt et al. 2002).

There was a high prevalence of MSC in the present study, with over 50% of the study population reporting moderate or severe MSC in Tromsø 7. This high prevalence could indicate that any MSC includes complaints that are more of a nuisance; not MSC that have a serious impact on quality of life. In this regard, severe MSC or having pain in ≥ 3 regions are likely more discriminant. In Tromsø 7, the prevalences of severe MSC and MSC in ≥ 3 regions were 11% and 18%, respectively. Nevertheless, pain is a strong risk factor for more pain, and even moderate pain is associated with a lack of labour force participation and absenteeism (Bergman et al. 2002; Elliott et al. 2002; Langley et al. 2010).

Self-reported working in a cold environment ≥ 25% of the time is an imprecise measure of cold exposure. Even though the question used to assess exposure specified outdoors, cold stores, or industry buildings, participants might have answered that they worked in a cold building simply because they considered their office to be cold. Some participants with occupations that are most likely performed in an office reported working in a cold environment, i.e. executive officers and customer service officers in banking (Supplementary Table 3). Consequently, we are at risk of classifying participants with minimal exposure to cold environments as exposed, which may have led us to underestimate the effect of working in a cold environment. On the other hand, our previous cross-sectional analysis showed that feeling cold was strongly associated with chronic pain (Farbu et al. 2019). Thus, the measure of cold exposure in this study might, to some degree, represent perceived thermal stress or an underlying trait that increases the likelihood of both feeling cold and developing MSC. Thus, misclassification of exposure might lead to both over- and underestimation of the effect. It should be mentioned that the high number of child-care workers who reported working in a cold environment is plausible, as most kindergarten classes in Norway spend some hours outdoors every day (Supplementary Table 3).

### Plausible causal pathway

Few plausible causal pathways between cold environments and MSC have been suggested in the literature. One possible pathway is the acute effects of cold environments on physiological function. Indeed, the capacity of a muscle to develop force and contraction velocity are reduced as muscular temperature lowers (Racinais and Oksa 2010), and increased co-activation of antagonist muscle can also occur, indicating poorer neuromuscular performance in cold environments. Moreover, the nerve conduction rate decreases, the elasticity of the tendons is decreased (Alegre et al. 2016), and if sufficiently cooled, the viscoelastic properties of the synovial fluid increase and make joints stiffer (Parsons 2014). All these changes increase the strain on the musculoskeletal apparatus and could increase the risk of overuse injuries like tendinopathy, as has been observed in earlier research (Kurppa et al. 1991; Milgrom et al. 2003). Other acute physiological changes could play a role as well; for example, increased muscle activity to produce heat will increase the load to the muscles and vasoconstriction following cold exposure could limit the distribution of important nutrients to cells not involved in thermogenesis (Parsons 2014).

It could be hypothesised that cold exposure contributes to a sensitisation process. A study of Danish slaughterhouse workers found that those who had the most complaints about the indoor climate, including cold temperature and draught, had a lower pressure pain threshold (Sundstrup et al. 2015).

### Strengths and limitations

One major strength of this study is its prospective design. Another strength is the reasonably high participation rate of Tromsø 6 and Tromsø 7 (65% in both), which increases the likelihood that the surveys contain a representative sample of the working population. There was a larger proportion of participants working in a cold environment ≥ 25% of the time who were lost to follow-up. Further, in a Norwegian cohort study, common health complaints, such as depression and musculoskeletal pain, increased the likelihood of participation in Tromsø 7 (Langhammer et al. 2012), which could have biased our results. However, the difference in loss to follow-up was not evident after exclusion of those with missing values in Tromsø 6. Thus, the analyses of the imputed datasets are more likely to be biased by loss to follow-up.

Individuals working in a cold environment tend to have more physically demanding work. Other known occupational risk factors for musculoskeletal pain could be unevenly distributed as well, i.e. poor posture or repetitiveness (Neupane et al. 2013). Therefore, our main analyses could be confounded. However, adjusting for occupational codes in the sensitivity analyses did not alter the strength of the association (Supplementary Table 2), making residual confounding by occupational factors less likely. On the other hand, it is possible that even within the same occupational code, those working in a cold environment ≥ 25% of the time are exposed to a different set of risk factors than those working in a cold environment < 25% of the time.

As the question about working in a cold environment was not repeated in Tromsø 7 we do not know if exposure was consistent between Tromsø 6 and Tromsø 7. Differences between exposed and non-exposed participants could have increased the probability of them changing occupations or exiting the work force, either due to health or changes in the labour market. If working in a cold environment in Tromsø 6 caused or aggravated pain, it is possible that some participants ended their exposure to reduce the risk of pain before Tromsø 7. In addition, the youngest participant in Tromsø 6 was 32 years of age, thus participants might have been exposed for over 10 years before even entering the study, and those most easily affected by a cold environment might already have developed MSC in Tromsø 6. Furthermore, they might have changed occupation prior to Tromsø 6 to reduce their exposure, and in turn, their risk of developing or aggravating existing MSC. Consequently, we might have underestimated the possible effect of cold environment due to the healthy-worker effect.

There are several diseases that probably increase the risk of developing MSC (Treede et al. 2015). These could be unevenly distributed between those working in a cold environment ≥ 25% of the time and those that do not. The lack of adjusting for these conditions is a limitation. However, we adjusted for self-reported health, which is thought to be a very inclusive measure of health (Mackenbach et al. 2002). Further, the origin of pain can be difficult to determine, and even though the participants were asked for pain and/or stiffness in muscles or joints, we cannot be sure that the complaints did not have other origins (Treede et al. 2015).

Tromsø is situated at 69° North, but has a moderately cold climate due to the Gulf Stream. The cold exposure is dependent on many factors other than ambient air temperature, i.e. amount of clothing or contact with cold surfaces or liquids. Thus, we expect that the results are relevant for other workers that are at risk of cold stress.

### Conclusion

Working in a cold environment ≥ 25% of the time increased the risk of future MSC. The increased risk was small, 15% after adjustment for possible confounders. There was no significantly increased risk of MSC in ≥ 3 regions, and no increased risk of severe MSC. However, the crude exposure measurement and the healthy worker effect might have biased the results. There is a need for prospective studies with a more precise measure of exposure.

## Electronic supplementary material

Below is the link to the electronic supplementary material.

Supplementary file1 (pdf 613KB)

## Data Availability

All statistical analyses were performed in Stata MP 15.

## Data availability

All data are available by applying to the Tromsø Study https://tromsoundersokelsen.uit.no/tromso
