# Supplementary material for: Is working in a cold environment associated with musculoskeletal complaints 7–8 years later? A longitudinal analysis from the Tromsø Study
Source: Int Arch Occup Environ Health. 2020 Nov 23;94(4):611–9. doi: 10.1007/s00420-020-01606-6 (PMC8068634; doi:10.1007/s00420-020-01606-6)
Supplement: Supplementary file 1 — Supplementary file1 (pdf 613KB) [file 420_2020_1606_MOESM1_ESM.pdf]

Supplementary tables

Supplementary table 1 Incidence rate ratio's (IRR) and confidence intervals (CI) from Poisson regression on the imputed data.

	Working in a cold environment <25% of the time n=4247	Working in a cold environment ≥25% of time n=632					
		Crude[a]			Fully adjusted[b]		
	Ref.	IRR	CI		IRR	CI	
Any MSC	-	**1.14**	**1.05**	**1.23**	**1.12**	**1.03**	**1.22**
Severe MSC	-	1.14	0.82	1.60	0.98	0.69	1.39
MSC in ≥3 anatomical regions	-	**1.28**	**1.06**	**1.56**	1.07	0.88	1.30

[a] Adjusted for age, sex, and number of MSC in Tromsø 6. Interaction term between working in a cold environment ≥25% of time and severe MSC/MSC in ≥3 anatomical regions.

[b] Adjusted for age, sex, number of moderate MSC, education, physical activity at work, smoking status, body mass index, and self-reported health at Tromsø 6. Interaction term between working in a cold environment ≥25% of time and severe MSC/MSC in ≥3 anatomical regions.

MSC=musculoskeletal complaints

Supplementary table 2 Sensitivity analysis for any musculoskeletal complaints (MSC), severe MSC, and MSC in ≥3 anatomical regions. Three different approaches are shown: a logistic regression, logistic regression adjusted for the 10 major occupational groups in the Norwegian version of the International Standard Classification of Occupations 88 (ISCO-88), and a model with a random intercept for each 4-digit occupational code. The odds ratio (OR), confidence intervals (CI) and Akaike information criterion (AIC) are shown for each model.

n=1982	Crude[a]				Fully adjusted model[b]			
	OR	CI		AIC	OR	CI		AIC
Any MSC								
Logistic model	**1.69**	**1.22**	**2.32**	2578	**1.79**	**1.23**	**2.60**	2566
Logistic model adjusted for the 10 major occupational groups	**1.52**	**1.07**	**2.16**	2589	**1.73**	**1.17**	**2.54**	2579
Mixed-effects logistic model with 4-digit occupational code	**1.66**	**1.20**	**2.31**	2580	**1.79**	**1.22**	**2.61**	2568
Severe MSC								
Logistic model	1.46	0.90	2.36	1079	1.18	0.66	2.12	1065
Logistic model adjusted for the 10 major occupational groups	1.03	0.60	1.76	1082	1.06	0.58	1.95	1070
Mixed-effects logistic model with 4-digit occupational code	1.46	0.90	2.36	1079	1.18	0.66	2.12	1065
MSC in ≥3 anatomical regions								
Logistic model	**1.71**	**1.16**	**2.51**	1725	1.43	0.93	2.21	1714
Logistic model adjusted for the 10 major occupational groups	**1.69**	**1.17**	**2.43**	1725	1.43	0.93	2.21	1714
Mixed-effects logistic model with 4-digit occupational code	1.40	0.93	2.10	1724	1.45	0.93	2.27	1722

[a] Adjusted for age and number of moderate MSC at Tromsø 6.

[b] Adjusted for age, number of moderate MSC, education, physical activity at work, smoking status, body mass index, and self-reported health at baseline.

Supplementary table 3 Occupations for which participants reported to work 25% of the time in cold environment at the time of Tromsø 6. Sorted by frequency of workers in the occupation.

Occupation	Working in a cold environment ≥25% of the time<br>Yes
Child-care workers	21
Carpenters and joiners	13
Stock clerks	10
Road workers and construction workers	10
Earth-moving and related plant operators	8
Mail carriers and sorting clerks	7
Fishery workers	7
Ships deck officers and pilots	6
Fish-processing-machine operators	6
Pre-primary education teaching associate professionals	5
Electricians, electrical and electronic equipment mechanics and fitters	5
Civil engineering technicians	4
Helpers and cleaners in offices and other establishments	4
Directors and chief executives	3
Nursing assistants and care	3
Shop salespersons and other salespersons (retail)	3
Dairy and livestock producers	3
Welders	3
Motor vehicle mechanics and fitters	3
Aircraft engine mechanics and fitters	3
Electrical line installers, repairers, and cable jointers	3
Heavy truck and lorry drivers	3
Shipsdeck crews	3
Labourers in construction and maintenance, etc.	3
Officers (above the rank of captain)	2
Other public service administrative associate professionals	2
Police officers	2
Clerical officers	2
Caretakers	2
Bricklayers and stonemasons	2
Concrete workers and site labourers	2
Telegraph and telephone installers and servicers	2
Bus- and tram drivers	2
Garbage collectors and related labourers	2
Production and operations department managers in manufacturing, mining and quarrying, electricity, gas and water supply	1
Production and operations department managers in education, health and social security	1
Production and operations department managers in personal care, cleaning and related services	1
Supply and distribution department managers	1
Other department managers not elsewhere classified	1

Transport clerks	1
General managers in construction	1
General managers in wholesale and retail trade	1
General managers in business services	1
Mining engineers, metallurgists, and related professionals	1
Biologists, botanists, zoologists, and related professionals	1
Nursing and midwifery professionals	1
Secondary education teaching professionals	1
Other public service administrative professionals	1
Electrical engineering technicians	1
Electronics and telecommunications engineering technicians	1
Oil, mining and metallurgical technicians	1
Fire inspectors	1
Nurses	1
Primary education teaching associate professionals	1
Estate agents	1
Technical and commercial sales representatives	1
Appraisers, valuers, and auctioneers	1
Bank associate professionals	1
Athletes, sportspersons and coaches	1
Radio and television announcers	1
Transport clerks	1
Dentists secretaries	1
Fire-fighters	1
Salespersons (wholesale)	1
Fish farmers, etc.	1
Plumbers	1
Painters and related workers	1
Chimney sweepers	1
Tinsmiths, etc.	1
Industrial mechanics and fitters	1
Technical illustrators	1
Oil- and gas-processing-plant operators	1
Fishing tackles-machine operators	1
Car, taxi, and van drivers	1
Labourers in manufacturing	1
Storing and goods handling labourers	1
